# Myotonia Congenita: Clinical Characteristic and Mutation Spectrum of CLCN1 in Chinese Patients

**DOI:** 10.3389/fped.2021.759505

**Published:** 2021-11-01

**Authors:** Chaoping Hu, Yiyun Shi, Lei Zhao, Shuizhen Zhou, Xihua Li

**Affiliations:** Department of Neurology, Children's Hospital of Fudan University, Shanghai, China

**Keywords:** myotonia congenita, *CLCN1* gene, mutations, genotype, phenotype

## Abstract

**Background:** *CLCN1*-related myotonia congenita (MC) is one of the most common forms of non-dystrophic myotonia, in which muscle relaxation is delayed after voluntary or evoked contraction. However, there is limited data of clinical and molecular spectrum of MC patients in China.

**Patients and Methods:** Five patients with myotonia congenita due to mutations in *CLCN1* gene were enrolled, which were identified through trio-whole-exome sequencing or panel-based next-generation sequencing test. The clinical presentation, laboratory data, electrophysiological tests, muscular pathology feature, and genetic results were collected and reviewed. We also searched all previously reported cases of MC patients with genetic diagnosis in Chinese populations, and their data were reviewed.

**Results:** The median onset age of five patients was 3.0 years old, ranging from 1.0 to 5.0 years old, while the median age of admit was 5.0 years old, ranging from 3.5 to 8.8 years old. Five patients complained of muscle stiffness when rising from chairs or starting to climb stairs (5/5, 100.0%), four patients complained of delayed relaxation of their hands after forceful grip (4/5, 80.0%), all of which improved with exercise (warm-up phenomenon) (5/5, 100%). Electromyogram was conducted in five patients, which all revealed myotonic change (100%). Genetic tests revealed nine potential disease-causing variants in *CLCN1* gene, including two novel variants: c.962T>A (p.V321E) and c.1250A>T (p.E417V). Literature review showed that 43 MC Chinese patients with genetic diagnosis have been reported till now (including our five patients). Forty-seven variants in *CLCN1* gene were found, which consisted of 33 missense variants, 6 nonsense variants, 5 frame-shift variants, and 3 splicing variants. Variants in exon 8, 15, 12, and 16 were most prevalent, while the most common variants were c.892G>A (p.A298T) (*n* = 9), c.139C>T (p.R47W) (*n* = 3), c.1205C>T(p.A402V) (*n* = 3), c.1657A>T (p.I553F) (*n* = 3), c.1679T>C (p.M560T) (*n* = 3), c.350A>G (p.D117G) (*n* = 2), c.762C>G (p.C254W) (*n* = 2), c.782A>G (P.Y261C) (*n* = 2), and c.1277C>A (p.T426N) (*n* = 2).

**Conclusion:** Our results reported five *CLCN1*-related MC patients, which expanded the clinical and genetic spectrum of MC patients in China. Based on literature review, 43MC Chinese patients with genetic diagnosis have been reported till now, and variants in exon eight were most prevalent in Chinese MC patients while c.892G>A (p.A298T) was probably a founder mutation.

## Introduction

Myotonia congenita (MC), which contributed 75% of non-dystrophic myotonia (1), is characterized by delayed muscle relaxation after voluntary or evoked contraction. According to different inheritance pattern, MC is divided into its dominant form Thomsen's disease (OMIM 160800) and recessive form Becker's disease (OMIM 255700).

The typical clinic characteristic of patients with MC included delayed relaxation after contraction, percussion myotonia, and warm up phenomenon (myotonia relieved after repeated activity). MC is associated with dysfunction of the voltage-gated chloride channel CLC-1 in skeletal muscle, which is encoded by the *CLCN1* gene mapped to chromosome 7q35. CLC-1 is important in maintaining the resting membrane potential, and certain mutations in *CLCN1* cause the protein to malfunction, resulting in plasma membrane hyper-excitation in skeletal muscle tissue and the “myotonic runs” typically seen in the electromyograms of myotonic patients. *CLCN1* gene contains 23 exons, and more than 200 mutations in *CLCN1* have been linked to MC (2, 3), including insertion, deletion, splice mutation, and frame-shift mutation.

In China, there have been some case reports and a few case series referring to *CLCN1*-related MC since the first gene-identified case in 2011 (4). Data of the clinical and molecular spectrum of MC patients in China was lacking. In this study, we analyzed the clinical and genetic characteristics of five unrelated MC patients, also collected other 38 previously reported MC patients with probable disease-causing *CLCN1* variants, aimed to reveal the clinical phenotype and mutation spectrum of *CLCN1*-related MC in China.

## Patients and Methods

### Patients

Five patients with myotonia congenita due to *CLCN1* gene mutations were enrolled from the Department of Neurology, Children's Hospital of Fudan University in September, January 2011, and March 2021. The clinical presentation, laboratory data, electrophysiological tests, pathology feature, and genetic results were collected and reviewed.

Ethical approval for the study was obtained from the health authority ethical committee of Children's Hospital of Fudan University. All the blood samples were collected after obtaining verbal consent from the parents of each patient in compliance with the Declaration of Helsinki.

### DNA Isolation, Molecular Tests, and Analysis

Genomic DNA of the children and their parents was extracted from whole blood using a QIAamp DNA Blood Mini Kit (Catalog no. 51106). Nucleic acid preparation and high-throughput sequencing (whole-exome sequencing or next-generation sequencing based on a neuromuscular disorder panel) were performed according to standard protocols in the Clinical Laboratory Improvement Amendments (CLIA) compliant sequencing laboratory in Wuxi NEXTCODE (288 Fute Zhong Road, Waigaoqiao Free Trade Zone Shanghai 200,131, China CLIA ID 99D2064856). Exome capture was performed using an Agilent Sure Select Human All Exon 50 Mb Kit (Agilent Technologies, Santa Clara, CA, USA) followed by sequencing as 150-bp paired-end runs on an Illumina XTen (Illumina, San Diego, CA, USA) platform. Segregation of the *CLCN1* variant within the family was confirmed by Sanger sequencing on the ABI 3730 Genetic Analyzer (Applied Biosystems, Foster City, CA, USA).

Sequence data were mapped to the human reference genome (GRCh37/hg19). Variant calling used the Genome Analysis Toolkit Best Practices Pipeline (Version 3.2.2). Data filtering, variant prediction, and interpretation followed the ACMG guidelines (5) and those from our previous work (6). Variant analysis was based on the 1,000 Genomes database (http://www.internationalgenome.org/), the gnomAD (http://gnomad-sg.org/), and an internal database (more than 30 k samples). The variants were predicted by the online software platforms PolyPhen2.2 (http://genetics.bwh.harvard.edu/pph2/), SIFT (http://sift.jcvi.org/), and Mutation Taster (http://www.mutationtaster.org/).

### Muscle Biopsy

A muscle biopsy was conducted in case 2. Muscle samples were appropriately oriented and frozen as we previously reported (7). Cryostat sections (8–10 μm) were cut from transversely oriented muscle blocks. Staining was performed with hematoxylin and eosin, modified Gomori trichrome (MGT), succinate dehydrogenase (SDH), cytochrome c oxidase (COX), and oil red O.

### Literature Review

Previously reported cases of Chinese population with *CLCN1*-related myotonia congenita were identified through search in available national database and international public data such as PubMed. The clinical, biochemical, electrophysiological, and molecular data were obtained from the respective references, reviewed, and compared with those of our present cases.

### Statistical Analysis

The GraphPad Prism software (version 6.01) was used for statistical analysis. For variables distributed in a normal fashion, mean ± standard deviation was calculated. For non-normally distributed variables, medians were calculated. Mann–Whitney *U*-test was used to compare quantitative variables and Chi-square test to compare qualitative variables (Fisher's exact test was used to analyze for component proportion in groups). A value of *p* < 0.05 was considered statistically significant.

## Results

### Clinical Presentation, Biochemical, and Electrophysiological Results of Five Patients

Five unrelated patients were enrolled including four boys and one girl. The clinical presentation and electromyography (EMG) results are shown in Table 1 (case 1–5). All patients developed normally before onset, except case 5, who had a slightly delayed motor milestone. There was no family history. The median onset age of five patients was 3.0 years old, ranging from 1.0 to 5.0 years old, while the median age of admission was 5.0 years old, ranging from 3.5 to 8.8 years old. Five patients complained of muscle stiffness when rising from chairs or starting to climb stairs (5/5, 100.0%), and four patients presented with delayed relaxation of their hands after a forceful grip (4/5, 80%), all of which improved with exercise (warm-up phenomenon) (5/5, 100%). The muscle stiffness aggravated in the morning (1/6, 16.7%), coldness (1/6, 16.7%). Laboratory tests showed slightly elevated creatine kinase (CK) level (278 IU/L, normal range: 210 IU/L) in one patient (1/5, 20%). Electrocardiogram (ECG) was conducted in four patients and echocardiography (ECHO) in three patients, which all showed normal (100%). Nerve conduction study was conducted in five patients which showed normal, while needle EMG test showed remarkable myotonic burst in five patients (5/5, 100%) and myopathic change in one patient (1/5, 20%) (Table 3).

**Table 1:** The clinical presentation, laboratory, and electrophysiological finding and molecular results of *CLCN1*-mutated patients in China.

**Case**	**Sex**	**FH**	**Onset (years)**	**Admit (years)**	**Clinical presentation and signs**								**EMG**		**Variants of** ***CLCN1***			
					**LL**	**UL**	**FA**	**MW**	**WP**	**HM**	**PM**	**AG**	**Tonic**	**Myo**	**IP**	**Exon**		**Variants**
1	M	−	3.0	5.0	+	+	−	−	+	−	−	Morning	+	−	AR	1	Paternal	c.139C>T (p.R47W)
															AR	8	Paternal	c.892G>A (p.A298T)
															AR	15	Maternal	c.1657A>T (p.I553F)
2	F	−	4.0	6.0	+	+	−	−	+	GM	−	Cold	+	−	AD*	15	Paternal	c.1649C>T (p.T550M)
3	M	−	5.0	8.8	+	−	−	−	+	−	−	−	+	−	AR	8	Paternal	**c.962T>A (p.V321E)**
															AR	3	Maternal	c.350A>G (p.D117G)
4	M	−	1.0	3.6	+	+	−	−	+	−	−	−	+	−	AR	11	Paternal	**c.1250A>T (p.E417V)**
															AR	12	Maternal	c.1277C>A (p.T426N)
5	M	−	2.5	3.5	+	+	−	−	+	−	−	−	+	+	AR	6	Maternal	c.762C>G (p.C254W)
															AR	8	Paternal	c. 892G>A (p.A298T)
6 (8)	F	+	Child	51	+	−	−	−	+	−	−	Fatigue; stress	+	−	AD	8	NA	c.907T>C (p.W303R)
7 (8)	M	−	13.0	27.0	+	+	−	−	+	Limbs	−	Cold	+	+	Sporadic?	6	NA	c.762C>G (p.C254W)
8 (8)	M	−	Child	24.0	+	+	+	−	+	Limbs	Thenar	Cold	+	−	AR	16	Maternal	c.1876C>T (p.R626*)
															AR	13	Paternal	c.1408A>G (p.M470V)
9 (9)	M	+	8.0	24.0	−	+	−	−	+	−	−	−	+	−	AD	16	Paternal	c.1879A>C (p.T627P)
10 (10)	M	+	14.0	17.0	+	+	+	−	+	BB	+	−	+	−	AD	8	Paternal	c.892G>A (p.A298T)
11 (10)	M	−	9.0	26.0	+	+	+	−	+	BB	−	Cold	+	−	Sporadic?	17	NA	c.2169C>A (p.S723R)
12 (11)	M	+	12.0	42.0	+	+	+	−	+	GM	GM	Angry; warm	+	−	AR?	10	NA	c.1129C>T (p.R377*)
															AR?		NA	c.1887delC(p.630fs)
13 (12)	M	+	6.0	14.0	+	+	−	+	+	BB; GM	BB; GM	Stress	+	−	AR?	8	Paternal	c.950G>A (R317Q)
															AR?	11	Maternal?	c.1205C>T (p.A402V)
14 (4)	F	+	1.5	6.5	+	+	+	−	+	FM; GM	GM	Cold	+	−	AD	8	Paternal	c.892G>A (p.A298T)
15 (13)	F	+	12.0	14.0	+	+	−	−	+	BB; GM	BB; GM	Cold	+	−	AD*	8	Maternal	c.937G>A(A313T)
															AD	11	Paternal	c.1205CT (p.A402V)
16 (14)	F	−	14.0	34.0	+	+	−	+	+	SGM; BB; GM	−	Cold	+	+	AR	12	Parental	c.1277C>A (T426D)
17 (15)	M	−	14.0	17.0	+	+	+	−	+	BB	BB; thenar	Cold	ND	ND	AR	11	Maternal	c.1205C>T (p.A402V)
															AR	8	Paternal	c.896T>C (p.V299A)
18 (16)	M	−	10.0	21.0	+	+	+	+	−	NA	NA	Cold	+	−	AR	In12	Paternal	c.1401+1G>A
															AR	15	Maternal	c.1657A>T (p.I553F)
19 (17)	M	+	12.0	16.0	+	+	−	−	+	+	+	Cold	+	−	AD	8	Paternal	c.871G>A (p.E291K)
																In17		c.2172+4A>G
20 (17)	M	−	26.0	32.0	+	+	+	−	+	+	+	Cold	+	−	AD	8	Parental	c.1013G>A (p.R338Q)
															AD	1	Parental	c.139C>T (p. R47W)
21 (17)	M	+	1.0	4.0	+	+	−	−	+	+	+	Cold	+	−	AD	8	Paternal	c.892G>A (p.A298T)
22 (17)	M	+	9.0	17.0	+	+	+	−	+	+	+	Cold	+	−	AD	8	Paternal	c.892G>A (p.A298T)
23 (17)	M	+	15.0	29.0	+	+	+	−	+	−	+	Cold	+	−	AD	3	Maternal	C.350A>G (p.D117G)
24 (18)	M	−	NA	1.0	+			−	+	−	NA	NA	+	−	AR	7	NA	c.829T>C (p.C277R)
															AR	8	NA	c.1012C>T (p.R338*)
25 (18)	M	+	NA	5.0	+			−	+	+	NA	NA	+	−	AD	12	NA	c.1262insC
26 (18)	M	−	NA	5.0	+			−	+	+	NA	NA	+	−	AR	8	NA	c.892G> A (p.A298T)
															AR	16	NA	c.1872G>T (p.E624*)
27 (18)	F	−	NA	11.0	+			−	+	+	NA	NA	+	+	AR	12	NA	c.1389insT
															AR	19	NA	c.2330delG
28 (18)	M	−	NA	9.0	+			−	+	+	NA	NA	+	+	AR	8	NA	c.892G>A (p.A298T)
															AR	2	NA	c.214_215delAG
29 (18)	M	+	NA	9.0	+			−	+	+	NA	NA	+	+	AD	19	NA	c.2362C>T (p.Q788*)
30 (19)	F	+	5.0	6.5	+			−	+	FM; GM	+	Cold	+	−	AD	8	Paternal	c.892G>A (p.A298T)
31 (19)	F	+	0.5	8.0	+			−	+	FM; GM; DEL	+	Stress; cold	+	−	AR	7	Maternal	c.782A>G (P.Y261C)
															AD*	15	Paternal	c.1679T>C (p.M560T)
32 (20)	F	+	4.0	19.0	+	+	−	+	+	GM	NA	Stress; hunger; fatigue	+	−	AD*	7	Paternal	c.782A>G (p.Y261C)
															AD*	22	Paternal	c.2576G>A (p.G859D)
33 (20)	M	+	2.0	27.0	+	+	+	−	+	General	NA	Cold; stress; hunger; fatigue	+	−	AD	14	Maternal	c.1568G>A (p.G523D)
34 (20)	M	−	4.0	16.0	+	+	+	−	+	General	NA	Cold; stress; hunger; fatigue	+	−	AR	15	Parental	c.1679T>C (p.M560T)
35 (20)	M	+	1.0	27.0	+	+	+	−	+	General	NA	Cold; stress; fatigue	+	−	AD*	15	Paternal	c.1679T>C (p.M560T)
															AR	In19	Maternal	c.2364+2T>C
36 (20)	M	+	9.0	34.0	+	+	−	+	+	General	NA	Cold	+	−	AR	1	Paternal	c.139C>T (p.R47W)
															AR	5	Maternal	c.685G > A (p.V229M)
37 (21)	F	+	Child	41.0	+	+	+	−	+	Mild	Thenar	Cold	+	−	AR	2	Paternal	c.280G>T (p.D94Y)
															AR	5	Maternal	c.618C>A (p.Y206*)
38 (22)	M	−	12.0	19.0	+	−	−	−	+	+	+	−	+	−	AR	15	Paternal	c.1744 A>T (p.I553F)
															AR	15	NA	c.1750C>A (p.H555N)
39 (22)	M	+	7.0	14.0	+	+	−	−	+	BB; GM	+	Cold; stress	+	−	AD	22	Paternal	c.2617C>T (p.L844F)
40 (23)	NA	−	Adol	NA	NA	+	NA	NA	−	NA	+	NA	+	−	AD	8	Sporadic?	c.929C>T (p.T310M)
41 (23)	NA	+	Adol	NA	NA	+	NA	NA	+	NA	+	NA	−	+	AD	13	Sporadic?	c.1412C>T (p.S471F)
42 (23)	NA	−	10.0	NA	NA	+	NA	NA	−	NA	+	NA	+	−	AD*	13	Paternal	c.1444G>A (p.G482R)
43 (23)	NA	−	24.0	NA	NA	+	NA	NA	−	NA	+	NA	+	−	AR	15	NA	c.1723C>T (p.P575S)
															AR	17	NA	c.1931A>G (p.D644G)

DM, motor and mental development; FH, family history; LL, lower limb; UL, upper limb; FA, facial; MW, muscle weakness; WP, warm-up phenomenon; HM, muscle hypertrophy; PM, percussion myotonia; AG, aggravation; CK, creatine kinase; tonic/myo, myotonic or myopathic damage in EMG; IP, inheritance pattern; AD, automatic dominant; AR, automatic recessive; AD*, automatic dominant with incomplete penetrance; child, childhood; Adol, adolescence; BB, musculus biceps brachii; GM, gastrocnemius; FM, forearm muscle; SGM, the shoulder girdle muscles; DEL, deltoid muscle. AD*, automatic dominant with incomplete penetrance (which has been provided in Table 1). ?, possible inheritance pattern (genetic results of parents not available); bold, novel variants found in our 5 patients.

### Muscle Biopsy

**Figure 1:**
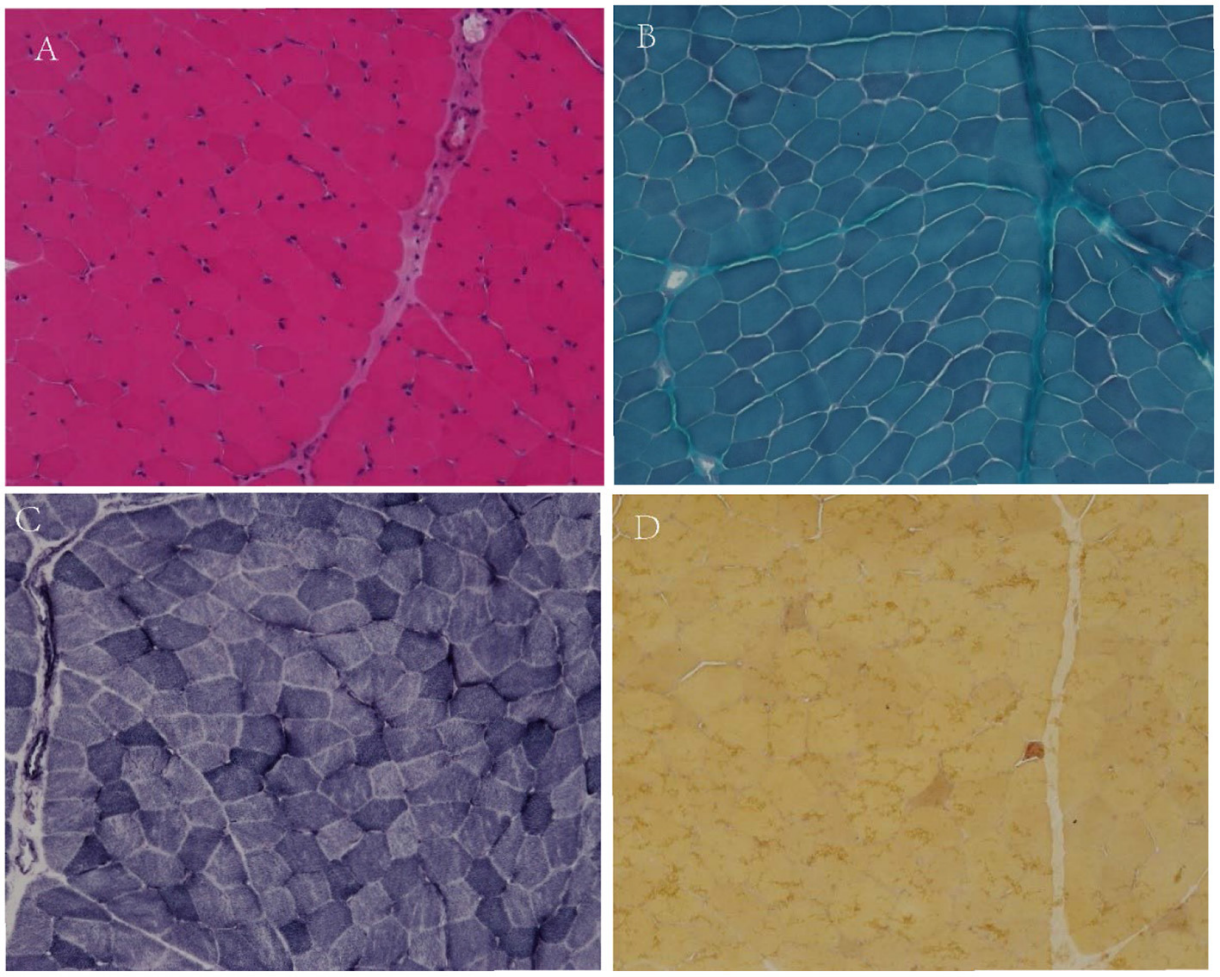
Muscular pathological results of patient 2. No variation in fiber size, no classical degeneration, necrosis, or regeneration in H&E staining (**A**, X200). No special hyperchromatic structures were observed in myofibrillar cytoplasm in MGT staining (**B**, X200). No abnormal fiber structures observed in NADH staining (**C**, X200). There were few angular muscle fibers in NSE staining (**D**, X200).

Muscle biopsy was conducted in the biceps brachii muscle of patient 2, which showed only few angular fibers, without variation in muscle fibers, degeneration, regeneration, internal nuclear, or enzymatic deficiency (Figure 1).

### Genetic Results

**Figure 2:**
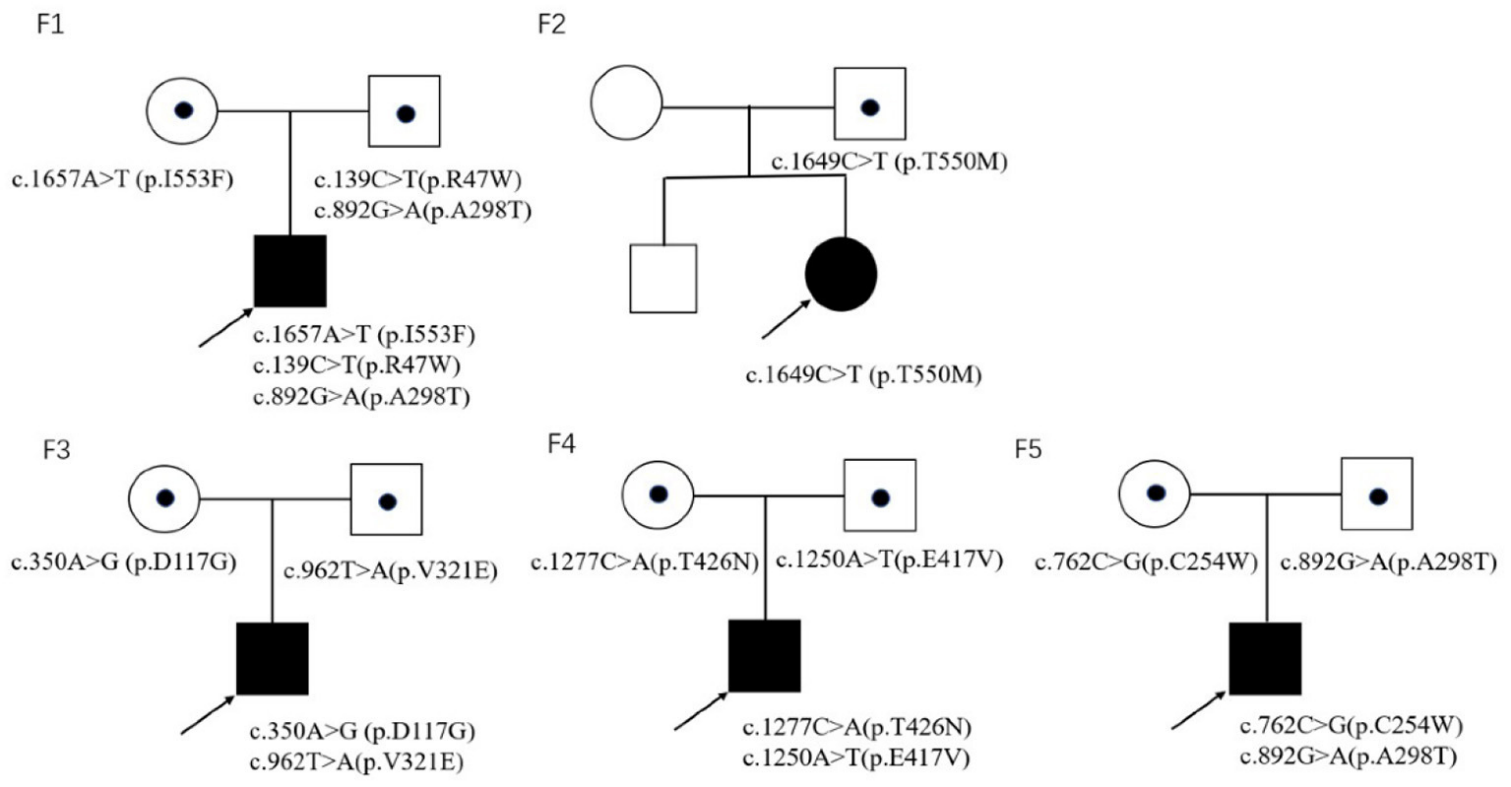
The pedigrees of five families in this study.

Trio-whole exome sequencing was conducted in case 1 and 3, while NGS-based genetic test of neuromuscular panel was performed in the rest of the patients. The mean depth of the trio-whole-exome sequencing data was ~120× . Molecular tests revealed nine potential disease-causing mutations in *CLCN1* gene (Figure 2), including seven reported variants: c.139C>T(p.R47W), c.892G>A (p.A298T), c.1657A>T (p.I553F), c.1649C>T (p.T550M), c.350A>G (p.D117G), c.762C>G (p.C254W), c.1277C>A (p.T426N), and two novel variants: c.962T>A (p.V321E) and c.1250A>T(p.E417V)(NM 000083), which have been submitted to the public database ClinVar (http://www.ncbi.nlm.nih.gov/clinvar, submission number: SUB 10199359).

In family 1, three reported pathogenic variants in the *CLCN1* gene were revealed in the proband, which were inherited from his parents, respectively.

In family 2, the proband was clinically diagnosed as myotonia congenita based on typical myotonia and tonic burst in EMG test (Table 3). Muscle biopsy excluded common metabolic myopathy and most congenital myopathy (Figure 1). Panel-based next-generation sequencing test revealed one reported pathogenic c.1649C>T (p.T550M) variant, which was inherited from his asymptomatic father who refused to receive physical examination and EMG test. Threonine residue is highly conserved, and c.1649C>T (p.T550M) is predicted to be probably damaging by software such as SIFT and Polyphen. Additionally, a previous experimental study has shown that it affects the gating of CLC-1 channels (24). Incomplete penetrance of this variant has been reported in some patients with a heterozygous state. Thus, we consider it to be pathogenic.

In family 3, the c.962T>A (p.V321E) variant has never been reported in the 1000 Genomes Project, once reported in the GnomAD database as heterozygous state, and is predicted to be probably damaging by software such as SIFT and Polyphen. According to the ACMG 2015 criteria (5), it is likely pathogenic (PM2 + PM3 + PP3 + PP4).

In family 4, the proband harbored compound heterozygous variants in *CLCN1* gene: a reported c.1277G>A (p.T426N) variant and a novel c.1250A>T (P.E417V) variant, which was inherited from his father and mother, respectively. The c.1250A>T (p.E417V) variant has never been reported in the 1000 Genomes Project, once reported in the GnomAD database as a heterozygous state, and is predicted to be probably damaging by software such as SIFT and Polyphen. According to the ACMG 2015 criteria (5), it is likely pathogenic (PM2 + PM3 + PP3 + PP4).

In family 5, molecular test in the proband revealed compound heterozygous variants in *CLCN1* gene, including a reported c.762C>G (p.C254W) variant and a reported c.892G>A (p.A298T) variant, which was inherited from his mother and father, respectively.

### Literature Review of Previously Reported Patients

Overall, 43 probands from unrelated families have been reported including our five patients (Table 1). The inheritance pattern of 14 patients was not clear because of invalid data of genetic results from the parents of the probands. Fourteen patients were classified as automatic dominant Thomsen's disease (14/29, 48.3%), while 15 patients were classified as automatic recessive Beck's disease (15/29, 51.7%).

The median onset age was 8 years old (ranging from 0.5 to 24 years old). The most common involved muscles with myotonia were lower limb muscle, followed by upper limb muscle and facial muscle. Warm-up phenomenon was observed in 93.1% patients and myotonic change in 100% patients. Creatine kinase was elevated in three patients (3/21, 9.7%), while cardiac problem was revealed in three patients (3/38, 7.9%) (Table 2).

**Table 2:** There was no significant difference in clinical presentation between AD and AR inherited MC patients in China.

	**Total (*n* = 29)**	**By inheritance pattern**		***p*-value**
		**AD (*n* = 14)**	**AR(*n* = 15)**	
Female	8/28 (28.6%)	4/13 (30.8%)	4/15 (26.7%)	>0.9999
Onset age	6 (0.5–26 years)	7.5 (1.0 > 26 years)	5 (0.5–14 years)	0.4214
LL myotonia	25/25 (100.0%)	11/11 (100.0%)	14/14 (100%)	>0.9999
UL myotonia	26/27 (96.3%)	13/13 (100.0%)	13/14 (92.9%)	>0.9999
FM myotonia	12/26 (46.2%)	6/12 (50.0%)	6/14 (42.9%)	>0.9999
Muscle weakness	5/28 (17.9%)	1/13 (7.7%)	4/15 (26.7%)	0.3333
Warm-up	27/29 (93.1%)	13/14 (92.9%)	14/15 (93.3%)	>0.9999
Muscle hypertrophy	21/27 (77.8%)	11/13 (84.6%)	10/14 (71.4%)	0.6483
Percussion myotonia	16/23 (69.6%)	10/12 (83.3%)	6/11 (54.5%)	0.1930
Myotonic change	28/28 (100.0%)	14/14 (100.0%)	14/14 (100.0%)	>0.9999
Myopathic change	2/14 (14.3%)	0/0 (0.0%)	2/14 (14.3%)	>0.9999

**Table 3:** The electrophysiological results of five patients in our study.

**Patient**	**Fibrillations**	**Positive sharp waves**	**Myotonic change**						**Motor unit potential (MUP)**	**Nerve conduction velocity**
			**FDI**	**BB**	**Deltoid**	**AT**	**GM**	**VM**	**Short polyphasic potentials**	
1	–	–	+	+	+	+	+	+	Normal	Normal
2	–	–	+	+	+	+	+	+	Normal	Normal
3	–	–	+	+	+	+	+	+	Normal	Normal
4	–	–	+	+	+	+	+	+	Normal	Normal
5	–	–	+	+	+	+	+	+	BB	Normal

*FDI, first dorsal interosseus; BB, Biceps brachii; AT, tibialis anterior muscle; GM, gastrocnemius; VM, vastus medialis*.

Overall, 47 variants of *CLCN1* gene in Chinese population were reported, which consisted of 33 missense mutations, 6 nonsense mutations, 5 frame-shift mutations, and 3 splicing mutations. Mutations in exons 8, 15, 12, and 16 were most prevalent, while the most common variants were c.892G>A(p.A298T) (*n* = 9), c.139C>T(p.R47W) (*n* = 3), c.1205C>T(p.A402V) (*n* = 3), c.1657A>T(p.I553F) (*n* = 3), c.1679T>C(p.M560T) (*n* = 3), c.350A>G(p.D117G) (*n* = 2), c.762C>G(p.C254W) (*n* = 2), c.782A>G(P.Y261C) (*n* = 2), and c.1277C>A(p.T426N) (*n* = 2).

## Discussion

Myotonia congenita is a non-dystrophic muscle disorder affecting the excitability of the skeletal muscle membrane, most of which are caused by mutations in the muscle chloride channel gene, *CLCN1* (25). Our study reported five Chinese patients with *CLCN1*-related myotonia, which revealed two novel likely pathogenic variants: c.962T>A (p.V321E) and c.1250A>T (p.E417V), which expanded the clinical and genetic spectrum of MC patients in the Chinese population.

**Figure 3:**
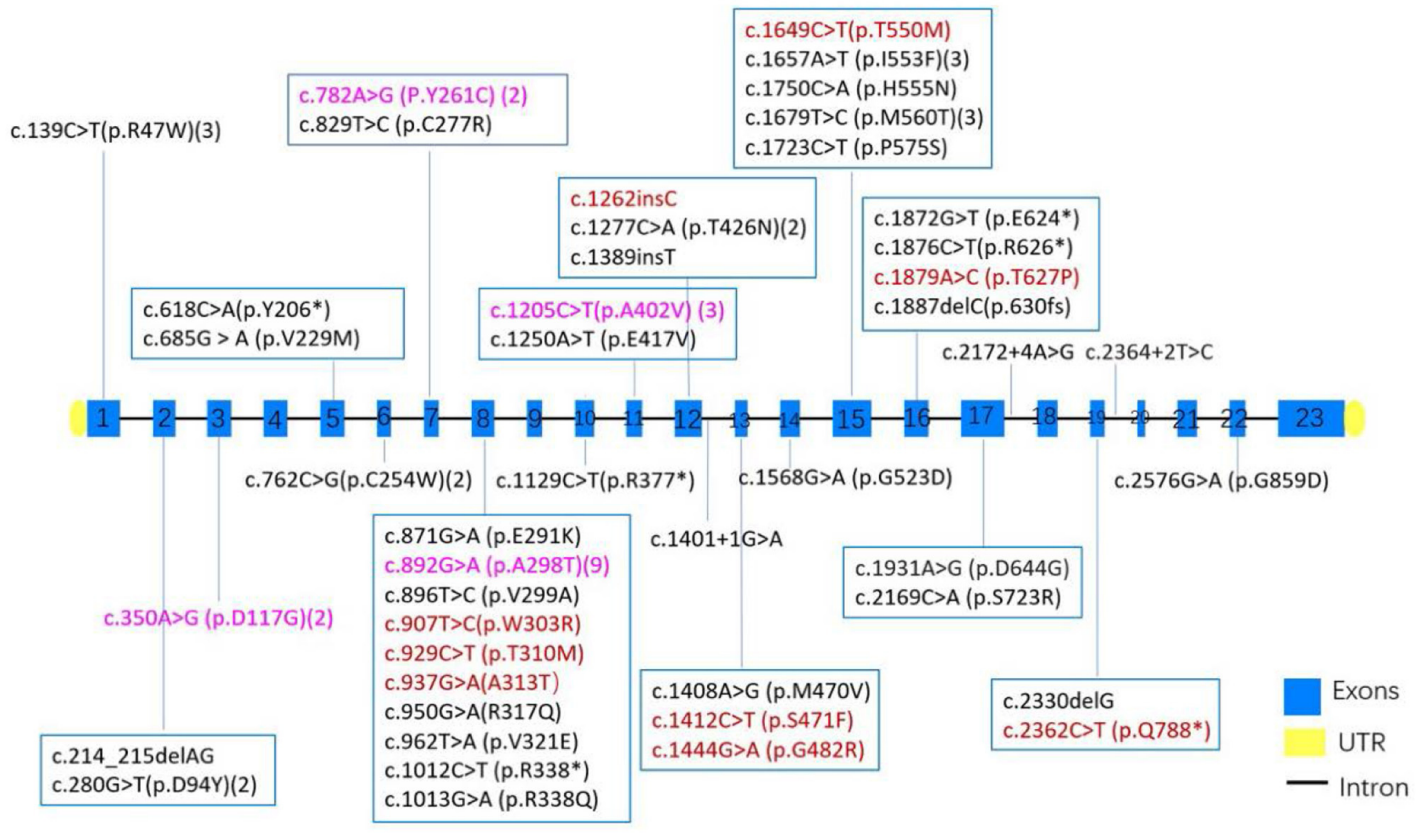
The distribution of exons and variants in 43 MC patients from China. Variants colored red have been observed to be AD inherited, black indicates AR inheritance, and purple suggests both AD and AR inheritance had been observed in the variants. Numbers after some variant showed the times of each variant found in 43 MC patients.

In our study, we found that in most pedigrees, the mutations co-segregated with myotonia; however, some did not. In family 2, he presented with typical myotonia and tonic discharge in the EMG test and inherited a reported pathogenic c.1649C>T (p.T550M) variant from his asymptomatic father. This variant has been observed in affected individuals in the heterozygous state with dominant transmission (26), with high clinical heterogeneity, ranging from occasional stiffness with EMG myotonic discharges finding to mild phenotype (24). It was possibly related with either reduced penetrance or incomplete dominance, which has also been observed in other variants including c.1444G>A (p.G482R) (23) or potential myotonia (27), but it also cannot be excluded that a second deep-intronic variant or small deletion may be missed in patients with a heterozygous state due to technical limitation. What is more, the c.1649C>T (p.T550M) variant has also been observed in combination with another *CLCN1* variant in several individuals affected with autosomal recessive myotonia congenita (28, 29). The same situation with alternating inheritance pattern occurred in four other variants including c.782A>G (P.Y261C), c.350A>G (p.D117G), c.1205C>T (p.A402V), and c.892G>A (p.A298T) (Figure 3). In our cohort, another noticeable phenomenon was that some probands harbored compound heterozygosity of two dominantly inheritable variants (case 15), which suggested the dosage effect of *CLCN1* mutation responsible for myotonia congenita of Thomsen type (30, 31). Sun pointed out that in 30–60% MC patients, the clinical presentation was not consistent with genetic results, which suggested the complexity of mechanism of MC (25), and the molecular mechanism of this complex genetic situation remains unclear.

Based on the review of all previously reported *CLCN1*-related MC patients in China, autosomal dominant Thomsen's disease compromised 48.3% of the patients, which was much higher than the percentage of 19–20% in one study (32), but lower than another report from Japan, which enrolled 30 families with myotonia congenita and showed that the dominant form consisted of 67% (27). Thus, it is possible that the dominant form of MC is more prevalent in Asia than in western countries.

In 24 families among all 47 variants in the *CLCN1* gene from 43 unrelated pedigrees, missense mutation was most common (33/47, 70.2%), followed by nonsense mutations (6/47, 12.8%), frame-shift mutations (5/47, 10.6%), and splicing mutations (3/47, 6.4%). Mutations in exons 8, 12, 15, and 16 were most prevalent in the Chinese population, contributing 35, 10, 9, and 7% of all variants, respectively, which is consistent with previous report that exon 8 of CLCN1 is a hot-spot for dominant mutations (32, 33).

In western countries, the c.2680C>T (p.R894*) variant is frequently found in Northern Europe especially in Germany (34), while c.180+3A>T was most frequent in Spain (35), c.1238T>G (p.F413C) in Netherlands (1), and c.501C>G (p.F167L) in Italy (36). In our cohort, the most common variants were c.892G>A (p.A298T), which contributed 20%, followed by c.1679T>C (p.M560T), c.139C>T (p.R47W), c.1205C>T (p.A402V), and c.1657A>T (p.I553F). This was, to some degree, consistent with one report in 2020, which pointed that A298T, P480T, T539A, and M560T mutations in *CLCN1* gene was common in Japan (27).

Until now, there are some medications for myotonia congenita patients, such as mexiletine and carbamazepine (37). However, there is still contradiction in the effectivity. Most patients in our cohort did not receive anti-myotonic medicines.

## Conclusion

Myotonia congenita is a clinical and genetic heterogenous disease. Our results expanded the clinical and genetic characteristics as well as identified mutation spectrum of MC patients in China. Mutations in exon 8 were most prevalent in Chinese MC patients, and c.892G>A (p.A298T) was probably a founder mutation.

## Data Availability Statement

The original contributions presented in the study are included in the article/Supplementary Files, further inquiries can be directed to the corresponding author/s.

## Ethics Statement

The studies involving human participants were reviewed and approved by the Health Authority Ethical Committee of Children's Hospital of Fudan University. Written informed consent to participate in this study was provided by the participants' legal guardian/next of kin. Written informed consent was obtained from the minor(s)' legal guardian/next of kin for the publication of any potentially identifiable images or data included in this article.

## Author Contributions

CH prepared and drafted this manuscript. YS conducted the electromyogram tests. SZ was responsible for the clinical genetic diagnosis. LZ took charge of the pathological analysis. XL fulfilled the data analysis and approved the submission of the manuscript. All authors contributed to the article and approved the submitted version.

## Conflict of Interest

The authors declare that the research was conducted in the absence of any commercial or financial relationships that could be construed as a potential conflict of interest.

## Publisher's Note

All claims expressed in this article are solely those of the authors and do not necessarily represent those of their affiliated organizations, or those of the publisher, the editors and the reviewers. Any product that may be evaluated in this article, or claim that may be made by its manufacturer, is not guaranteed or endorsed by the publisher.
