# Supplementary material for: Myotonia Congenita: Clinical Characteristic and Mutation Spectrum of CLCN1 in Chinese Patients
Source: Front Pediatr. 2021 Nov 1;9:759505. doi: 10.3389/fped.2021.759505 (PMC8591224; doi:10.3389/fped.2021.759505)
Supplement: Supplementary file 1 [file Table_1.DOCX]

Additional data: list of 167 genes included in the panel of neuromuscular disease

| ABHD5 | ACADL | ACADM | ACADS | ACADVL | ACTA1 | ADCK3 | AGK |
| --- | --- | --- | --- | --- | --- | --- | --- |
| AGL | AGRN | ALDOA | ALG14 | ALG2 | ANO5 | ATP2A1 | B3GALNT2 |
| B4GALT1 | B4GAT1 | BAG3 | BIN1 | BOLA3 | CACNA1S | CAPN3 | CAV3 |
| CCDC78 | CFL2 | CHAT | CHKB | CHRNA1 | CHRNB1 | CHRND | CHRNE |
| CHRNG | CLCN1 | CNTN1 | COL12A1 | COL6A1 | COL6A2 | COL6A3 | COLQ |
| COQ2 | COQ6 | COQ9 | CPT2 | CRYAB | DAG1 | DES | DMD |
| DNAJB6 | DNM2 | DOK7 | DOLK | DPAGT1 | DPM2 | DPM3 | DUX4 |
| DYSF | EMD | ENO3 | EPM2A | ETFA | ETFB | ETFDH | ETHE1 |
| FHL1 | FKRP | FKTN | FLNC | GAA | GBE1 | GFPT1 | GMPPB |
| GNE | GYG1 | GYS1 | HADH | HNRNPDL | HSPG2 | ISCU | ISPD |
| ITGA7 | ITGA9 | KBTBD13 | KCNA1 | KCNE3 | KCNJ16 | KCNJ18 | KCNJ2 |
| KCNQ1 | KLHL40 | KLHL9 | LAMA2 | LAMB2 | LAMP2 | LARGE | LDB3 |
| LDHA | LMNA | LPIN1 | LRP4 | MATR3 | MEGF10 | MSTN | MTM1 |
| MUSK | MYBPC1 | MYBPC3 | MYH2 | MYH3 | MYH7 | MYH8 | MYOT |
| NEB | NHLRC1 | ORAI1 | PABPN1 | PDSS1 | PDSS2 | PFKM | PGAM2 |
| PGK1 | PGM1 | PHKA1 | PLEC | PNPLA2 | POLG | POMGNT1 | POMGNT2 |
| POMK | POMT1 | POMT2 | PRKAG2 | PTRF | PYGM | RAPSN | RBCK1 |
| RYR1 | SCN4A | SEPN1 | SGCA | SGCB | SGCD | SGCG | SLC22A5 |
| SLC25A20 | SMCHD1 | STIM1 | SYNE1 | SYNE2 | TCAP | TIA1 | TK2 |
| TMEM5 | TNNC1 | TNNC2 | TNNI1 | TNNI2 | TNNT1 | TNNT3 | TNPO3 |
| TOR1AIP1 | TPM2 | TPM3 | TRIM32 | TTN | VCP | VMA21 |  |
